# Doping of casted silk fibroin membranes with extracellular vesicles for regenerative therapy: a proof of concept

**DOI:** 10.1038/s41598-024-54014-y

**Published:** 2024-02-12

**Authors:** Sandra Fuest, Amanda Salviano-Silva, Cecile L. Maire, Yong Xu, Christian Apel, Audrey Laure Céline Grust, Arianna Delle Coste, Martin Gosau, Franz L. Ricklefs, Ralf Smeets

**Affiliations:** 1 Department of Oral and Maxillofacial Surgery, Division of Regenerative Orofacial Medicine, University Medical Center Hamburg-Eppendorf, 20246 Hamburg, Germany; 2 Department of Neurosurgery, University Medical Center Hamburg-Eppendorf, 20246 Hamburg, Germany; 3 Department of Biohybrid and Medical Textiles (BioTex), AME - Institute of Applied Medical Engineering, Helmholtz Institute of RWTH Aachen University and Hospital, 52074 Aachen, Germany; 4 Department of Oral and Maxillofacial Surgery, University Medical Center Hamburg-Eppendorf, 20246 Hamburg, Germany

**Keywords:** Medical research, Materials science

## Abstract

Bioactive material concepts for targeted therapy have been an important research focus in regenerative medicine for years. The aim of this study was to investigate a proof-of-concept composite structure in the form of a membrane made of natural silk fibroin (SF) and extracellular vesicles (EVs) from gingival fibroblasts. EVs have multiple abilities to act on their target cell and can thus play crucial roles in both physiology and regeneration. This study used pH neutral, degradable SF-based membranes, which have excellent cell- and tissue-specific properties, as the carrier material. The characterization of the vesicles showed a size range between 120 and 180 nm and a high expression of the usual EV markers (e.g. CD9, CD63 and CD81), measured by nanoparticle tracking analysis (NTA) and single-EV flow analysis (IFCM). An initial integration of the EVs into the membrane was analyzed using scanning and transmission electron microscopy (SEM and TEM) and vesicles were successfully detected, even if they were not homogeneously distributed in the membrane. Using direct and indirect tests, the cytocompatibility of the membranes with and without EVs could be proven and showed significant differences compared to the toxic control (*p* < 0.05). Additionally, proliferation of L929 cells was increased on membranes functionalized with EVs (*p* > 0.05).

## Introduction

**Figure 1:**
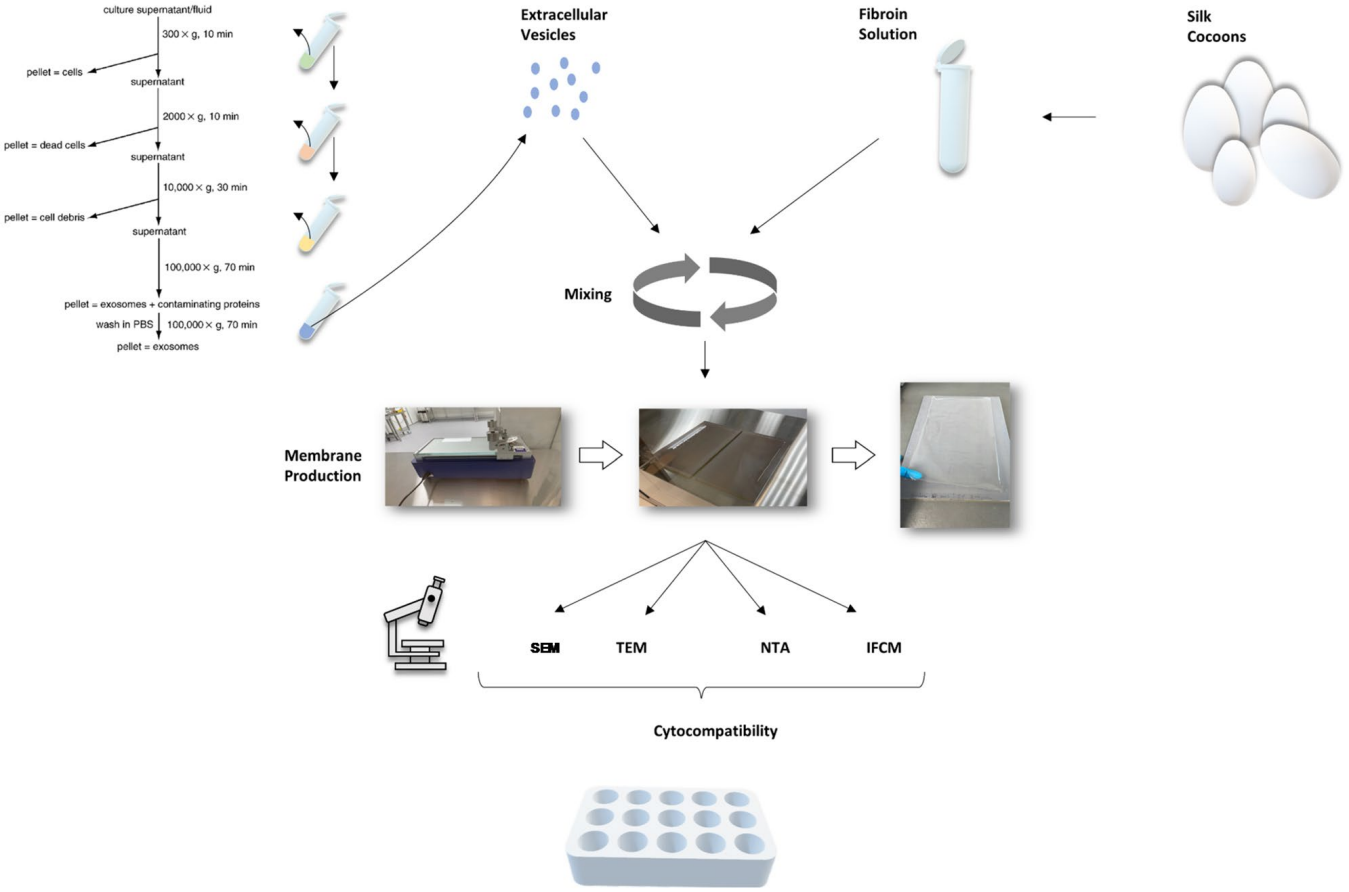
Schematic representation of the experiments carried out in this study.

Extracellular vesicles (EVs) have emerged in recent years as an important mediator of intercellular communication. They possess multiple capabilities to act on their target cells and can thus play critical roles in both physiology and pathology^1^. EVs carry complex biological information consisting of soluble and transmembrane proteins, RNAs and miRNAs, DNA, and lipids^2^. They can be taken up by recipient cells and release their bioactive contents, which can subsequently significantly alter the phenotype of the recipient cell. EVs released from mesenchymal stem cells have a similar therapeutic effect as the cells from which they are derived of^3,4^. Thus, they show remarkable effect on promoting regeneration on different types of tissues. The use of EVs has been shown to be more beneficial than the use of mesenchymal stem cells themselves for several reasons^2,4^. EVs are not limited by viability, cannot replicate, and therefore cannot degenerate. They also express limited major histocompatibility markers (MHC) and have immunomodulatory properties that reduce the risk of negative immune responses while preserving beneficial regenerative properties^5,6^. In addition, a key advantage is that unlike cells, EVs can be stored for longer periods of time^7^. Thus, they can be frozen and thawed or even lyophilized without major functional losses^8^. EVs are considered as promising in diagnostics and as new tools for various therapeutic approaches, including regenerative therapies and drug delivery, for anti-tumor therapy, and in the context of vaccination strategies^9–21^. The beneficial effects of EVs from mesenchymal stem cells on wound healing and tissue regeneration have already been demonstrated in various cell culture experiments and animal models, and were manifested by improved re-epithelialization, accelerated collagen deposition and angiogenesis among others^22–24^. In particular, EVs derived from dental pulp stem cells promote endothelial cell proliferation through the expression of proangiogenic factors and have hence been shown to drive angiogenesis^22,25^. Huang et al. demonstrated that EVs derived from dental pulp stem cells induce odontogenic differentiation both in vitro and in vivo^26^. Liu et al.^23^ further demonstrated the efficacy of EVs in periodontal tissue regeneration. As a result, there is a growing interest in exploring the use of EVs for regenerative treatments, particularly in the head and neck region. However, one of the major challenges for the therapeutic application of EVs is that free EVs are rapidly excreted in the bloodstream and ultimately digested by macrophages^27^. The clinically very relevant problem of keeping the EVs at the site of action for a longer period of time or enabling a controlled release of these can be achieved by immobilizing the EVs in a suitable carrier structure. Various research groups have already shown that the integration of EVs into different biomaterial-based carrier structures such as hydrogels, hydrogel patches, and 3D scaffolds proved to be more effective than direct injection of EVs into the defect^22,28–31^. As a novel approach, the biomaterial silk fibroin (SF), which is extracted from the cocoon of the mulberry silkworm *Bombyx mori* (*B. mori*), can be used as a carrier material. SF as a medical carrier material is increasingly becoming the focus of research for new biomedical applications, such as wound dressings, because silk fibroin is an ideal starting material due to the dissolution of silk fibers in an aqueous solution, which can be formed into almost any shape by incorporating numerous available textile techniques (e.g. weaving, knitting and braiding)^32–34^. The advantages of SF as a biomaterial are its high strength, moisture retention, flexibility, biocompatibility, bioresorbability, oxygen permeability, and hemostatic ability^33–36^. Furthermore, the degradation and release kinetics of silk fibroin products can be precisely controlled. Especially in the broad field of oral and maxillofacial surgery, the use of regenerative biomaterials after trauma, surgical procedures or aesthetic applications is a standard procedure. Bone substitute materials and collagen membranes for instance are used in the field of guided bone and soft tissue regeneration (guided bone regeneration (GBR)/ guided tissue regeneration (GTR)), which, however, still have material-specific shortcomings due to their manufacturing chain^37^. SF can be processed by the electrospinning method, in which fibers in the size range of micro- to nanometers are obtained from a polymer solution by applying an electrical force^33^. Nonwoven structures can also be fabricated in this way. Fibroin-based films, nonwovens and also 3D scaffolds are excellent for biomedical applications such as wound dressings due to their mechanical properties and porous structure, which promotes cell infiltration and vascular sprouting^32^. In the present study we tested the hypothesis that EVs can be successfully incorporated into SF-membranes during the production process. EVs were isolated from the supernatant of an immortalized Gingiva-Fibroblast (GF) cell line and analyzed by NTA and ICFM analysis and subsequently incorporated into the SF-membranes. Scanning electron microscopy was used to examine the surface structures as well as cross-sections of the membranes, and transmission electron microscopy was used to demonstrate evidence of EVs in the membranes. The membranes were further examined with regards to cytotoxicity and proliferation both macroscopically and in cell culture, see Fig. 1.

## Results

### Characterization of the EVs

**Figure 2:**
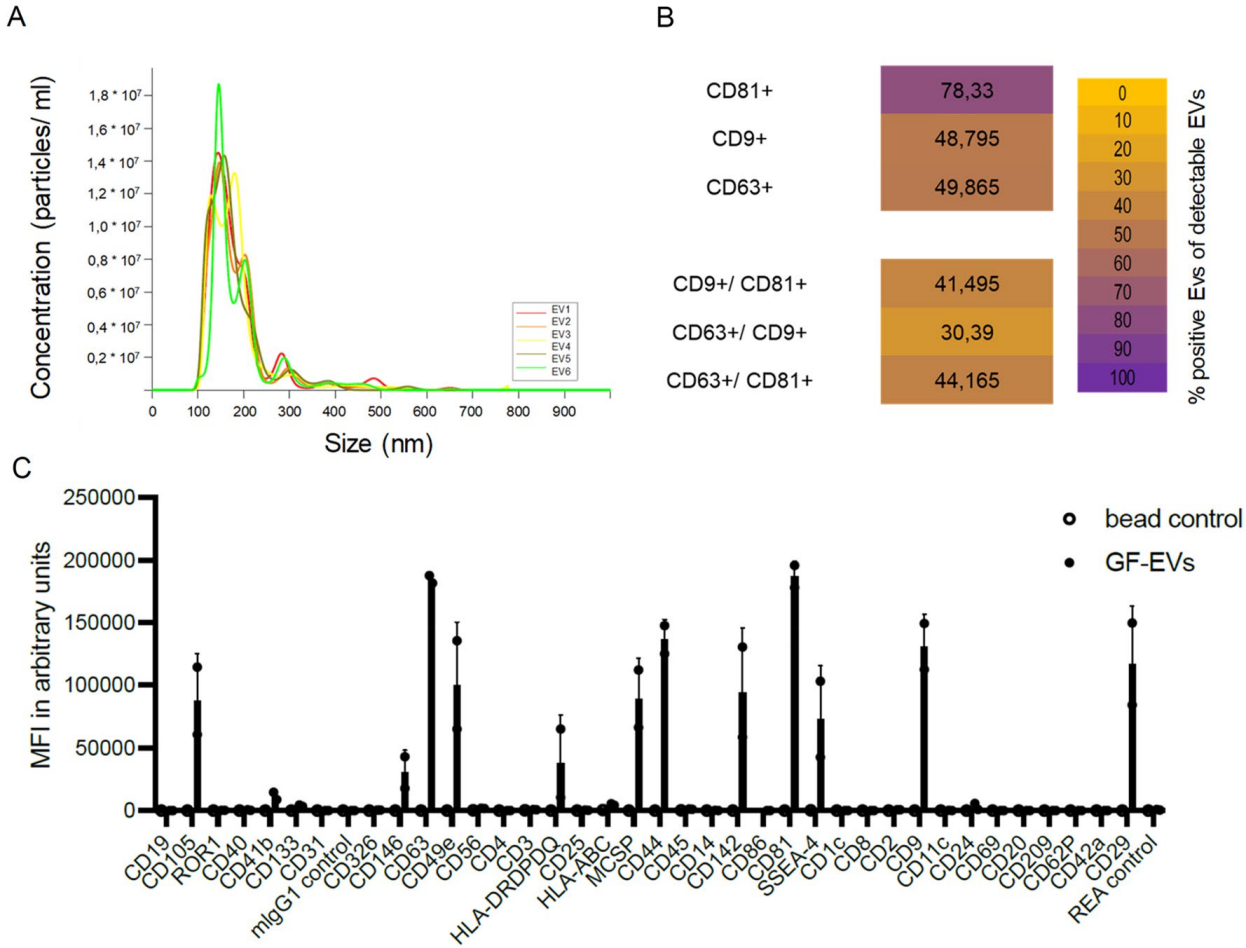
(**A**) Representative NTA Graph of six biological replicates (EV1–EV6); (**B**) IFCM analysis on EVs stained with antibodies against CD9, CD63 and CD81 described in^38^; (**C**) Phenotypical characterization of EVs using MACSPlex Exosome kit (Miltenyi) as described in the material and methods section.

The size distribution of EVs was determined by Nanoparticle Tracking Analysis (NTA), presented by six biological replicates (EV1–EV6) isolated from an immortalized gingiva-fibroblast cell line. The majority of EVs from gingvia fibroblasts, 88.9%, had a size below 250 nm with a peak at 147 nm, which falls within the particle size range of small EVs, previously known as exosomes. A second peak was observed at 289 nm, representing only 0.05% of EVs, and a mere 0.007% of EVs were above 500 nm (Fig. 2A). Additionally, the majority of secreted EVs exhibited high expression of common EV markers (i.e. CD9, CD63 and CD81), as characterized by Imaging Flow Cytometry (IFCM) (Fig. 2B). Out of those markers, CD81 showed the highest abundancy (78.33%) on gingiva-fibroblast EVs, a tetraspanine known for its importance of EV functionality, as it facilitates interactions with target cells and contributes to their role in intercellular communication and cargo delivery. To further analyze the molecular profile of gingival fibroblast-derived EVs, we employed a multiplex bead-based assay, enabling the simultaneous assessment of 39 surface proteins (Fig. 2C). In addition to confirming our IFCM results by detecting high levels of CD9, CD63 and CD81, this assay revealed elevated levels of CD105, CD29, CD142 and CD44 on our EVs. These markers, associated with diverse cellular processes, indicate the multifaceted nature of gingiva-fibroblast EV functions. The presence of CD105, CD29, CD142, and CD44 on EV surfaces suggest potential contributions to intercellular communications, tissue homeostasis, and responses to physiological cues. This comprehensive characterization enhances our understanding of the molecular cargo carried by gingival fibroblast-derived EVs and emphasizes their versatility in mediation various cellular processes.

### Structural analysis of the SF-membranes

**Figure 3:**
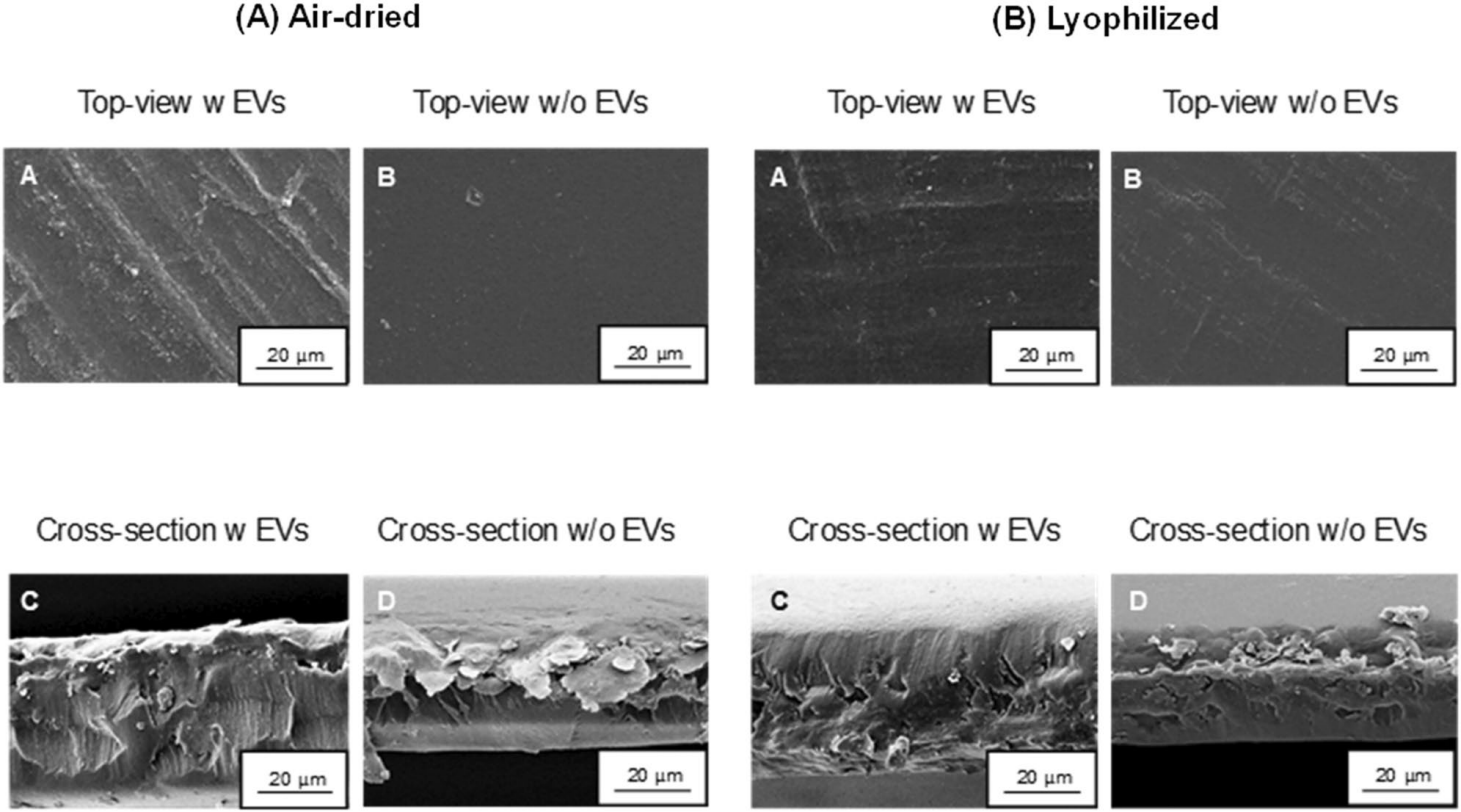
SEM-images of SF-membranes in 40 µm diameter with (w) and without (w/o) vesicles after different drying processes. (**A**) Air-dried membranes: top-view (**A**, **B**) and cross-section (**C**, **D**); (**B**) Lyophilized membranes: top-view (**A**, **B**) and cross-section (**C**, **D**) in 1000 × magnification.

Figure 3 demonstrates on the left side (A) the air-dried membranes with (w) (A, C) and without (w/o) (B, D) EVs in cross-section and top view. With the achieved resolution, no EVs in the form of round shape structures could be detected in the SF-substrate material. Both membranes show a homogeneous and amorphous surface structure, however, membrane A shows a more pronounced line structure than membrane B. In cross-section, both membranes seem to behave similarly. No pore structure can be visualized, the membrane with EVs shows many small particles in the cross section, which are assumed to be fibroin residues due to their size. The membrane without EVs (D) presents as flat and homogeneous in the core, the outer regions however show scale-like fibroin residues. The right side of figure B shows the same arrangement as in A, with the difference that the membranes were freeze-dried to better display EVs. Again, homogeneous amorphous SF structures were clearly revealed in the top-view, representing a uniform membrane structure. In cross-section, the membranes appear to be less SF-particulate in contrast to the air-dried membranes. Especially the outer regions show smooth structures. Further information on EVs in the SF-drawn membranes cannot be made from these images.

**Figure 4:**
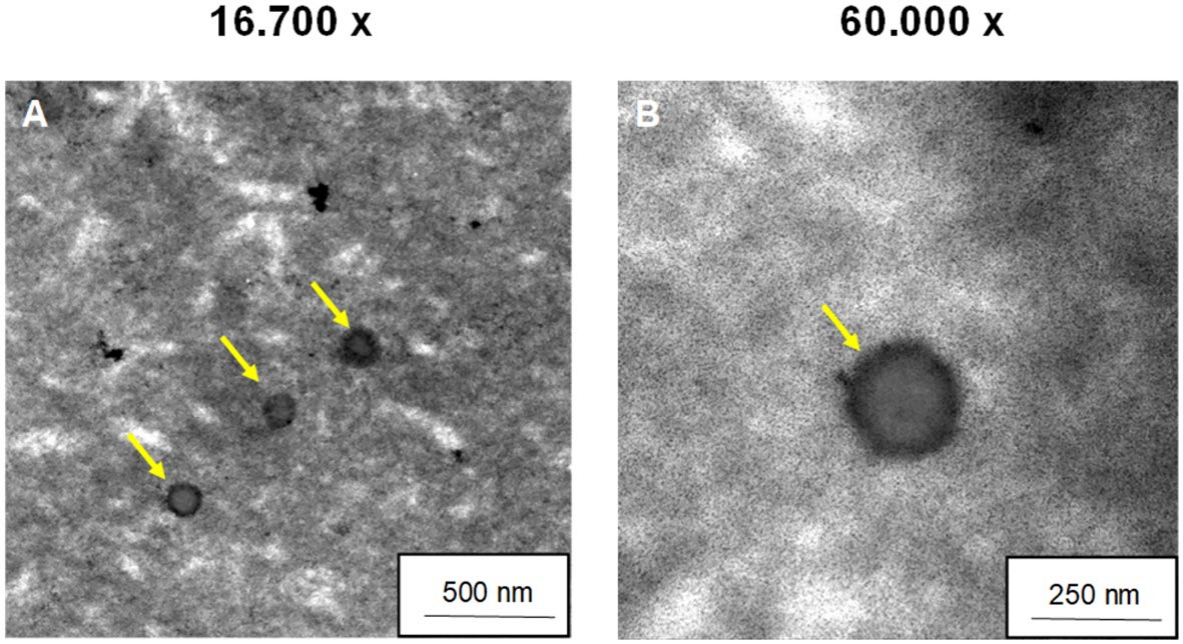
TEM-images of SF-membranes w EVs in 16.700 x (**A**) and 60.000 x (**B**) magnification.

Close inspection of the TEM images in Figure A and B reveals EVs at both 16.700 × magnification and 60.000 × , see Fig. 4. The yellow arrows visualize the detection of EVs. The vesicles in the form of small round spheres with a darker rim and lighter core are not homogeneously distributed in the SF membrane.

### Cell proliferation assay (XTT)

**Figure 5:**
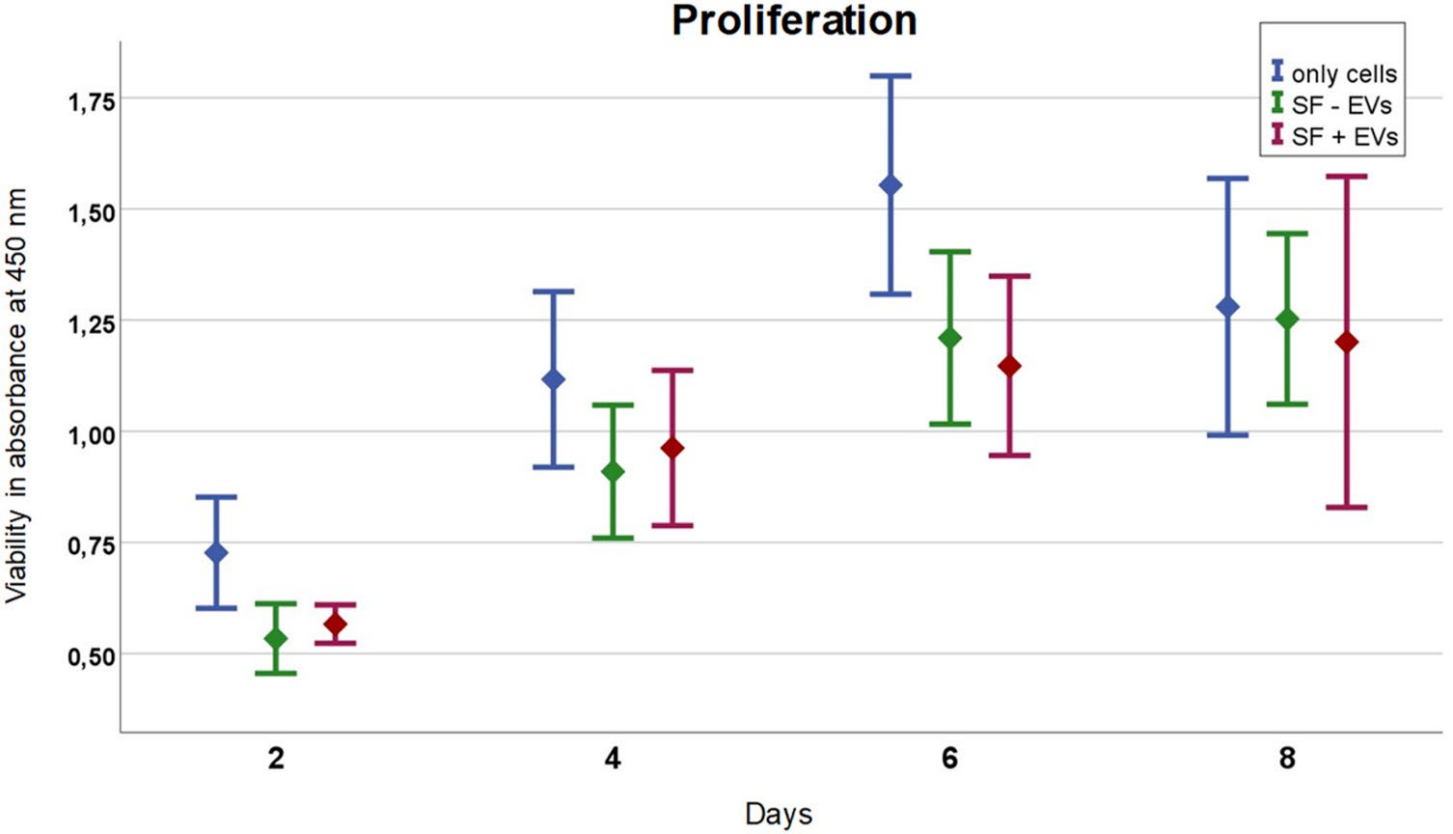
The Proliferation-Assay was performed on L929 cells treated with SF w EVs and SF w/o EVs and only cells. The cell proliferation was characterized using XTT on 2, 4, 6 and 8 days. Data are presented as mean ± SD.

In this study, we performed a XTT-Assay on L929 cells to assess cell viability and metabolic activity on different days (2, 4, 6 and 8 days (d)), see Fig. 5. These cell lines are commonly used due to their availability and relevance for various research areas such as toxicology, pharmacology, and cell biology. By measuring the metabolic activity of these cells, researchers can evaluate the impact of treatments, test substances, or drugs on their viability and overall functionality. The XTT assay serves as a valuable tool for proliferation, drug screening, and understanding cellular responses to different experimental conditions.

The number of cells in the experimental group with EVs was higher after five days than in the control group without EVs. From day 5, the non-functionalized membrane proliferates better and peaks on day 8. The membrane loaded with EVs also peaks on day 8, but to a lesser extent than the control membrane. There were no statistical differences among the experimental groups. The control group consisting of pure cells proliferates much better than the two membrane types, but reaches its peak already on day 6. However, it is important to note that while the XTT assay provides a valuable indication of cell viability, additional experiments and analyses, such as cytotoxicity assays and molecular investigations, should be performed to validate and better understand the underlying mechanisms of the observed protective effects.

### Lactate dehydrogenase assay (LDH)

An LDH assay was also performed on L929 and MC3T3 cells to allow for the evaluation of cytotoxicity, cell damage, comparative analysis, and drug screening. This assay provides valuable insights into the effects of various treatments or interventions on cell viability and membrane integrity, aiding in understanding cellular responses and informing the development of potential therapies.

**Figure 6:**
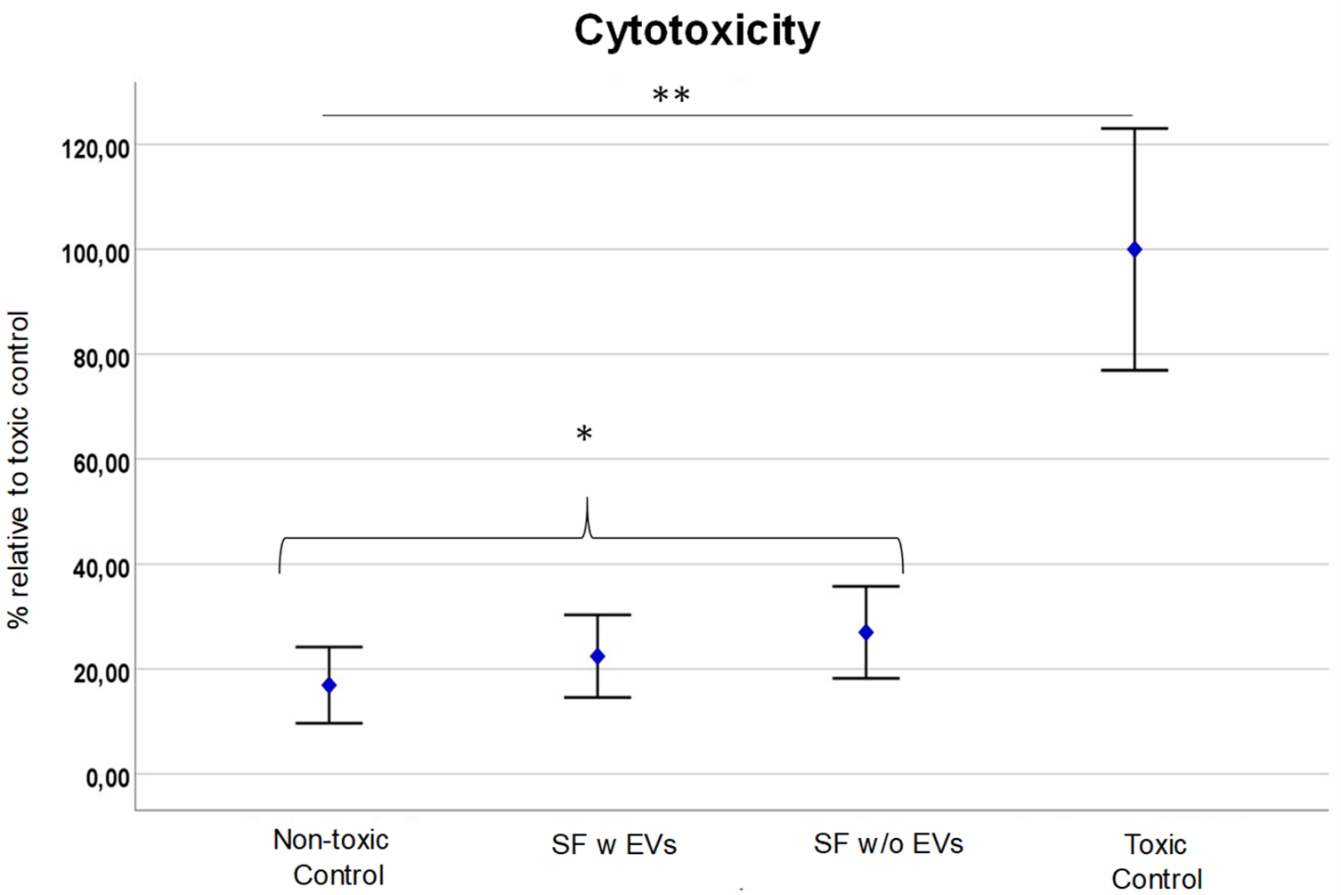
LDH activity in L929 cells treated with SF w EVs and SF w/o EVs, toxic control, and non-toxic control. Data is represented as mean ± standard deviation (SD) of three independent experiments. Statistical significance was determined using one-way analysis of variance (ANOVA) followed by LSD post-hoc test. *p > 0.05 indicates non statistic significances between the tested groups; and **p < 0.05 indicates a significant difference compared to the toxic control group.

**Figure 7:**
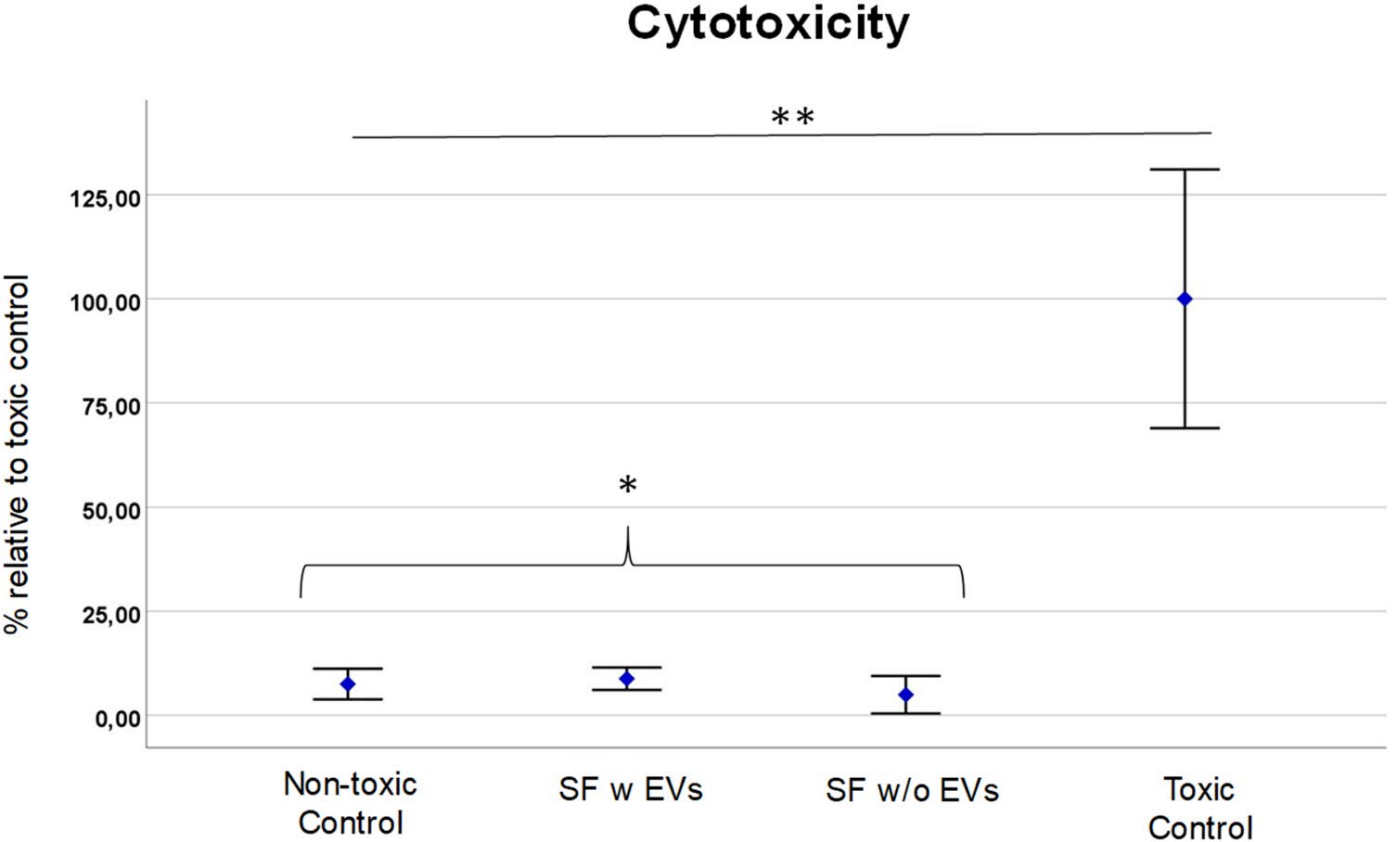
LDH activity in MC3T3 cells treated with SF w EV, SF w/o EVs, toxic control, and non-toxic control. The results demonstrate that both SF w EVs and SF w/o EVs induced a slightly higher LDH activity compared to the non-toxic control group, suggesting a mild toxicity effect. However, LDH activity was significantly lower in both samples than the toxic control group, indicating their non-toxic nature. Statistical significance was determined using one-way analysis of variance (ANOVA) followed by LSD post-hoc test. *p > 0.05 indicates non statistic significances between the tested groups; **p < 0.05 indicates a significant difference compared to the toxic control group.

We could report SF w EVs and SF w/o EVs exhibited slightly higher cell toxicity on L929 cells compared to MC3T3 cells, as indicated by the LDH assay results. In the LDH assay, both SF w EVs and SF w/o EVs demonstrated significantly higher viability compared to the toxic control group (*p* < 0.05). In L929 cells, both samples showed a modest but not statistically significant increase in LDH activity compared to the corresponding non-toxic control group (*p* > 0.05) (Fig. 6). This suggests a mild toxic effect of SF w EVs (MV = 22.4540; SD = 9.42404) and SF w/o EVs (MV = 27.0078; SD = 10.49305) on L929 cells. However, a trend in the LDH assay on L929 cells can be identified which shows that SF w EVs achieves a lower toxicity than SF w/o EVs. Due to the small number of cases, this hypothesis can however not be proven substantially. Interestingly, when tested on MC3T3 cells, SF w EVs and SF w/o EVs displayed a relatively lower LDH activity, indicating a reduced toxic impact compared to L929 cells (Fig. 7). Nevertheless, this difference did not reach statistical significance (*p* > 0.05). In general, these findings suggest that L929 cells may be more susceptible to the toxic effects of SF w EVs and w/o EVs compared to MC3T3 cells. The different responses in cell toxicity between L929 and MC3T3 cells may be attributed to inherent variations in cellular characteristics, sensitivity, or metabolic processes. Further investigations are required to elucidate the underlying mechanisms responsible for the observed differences in toxicity profiles. These results highlight the importance of considering cell line-specific responses in assessing cellular toxicity and understand the need for comprehensive toxicity evaluations using multiple cell lines to ensure robust and reliable conclusions.

### Live/ Dead-staining

**Figure 8:**
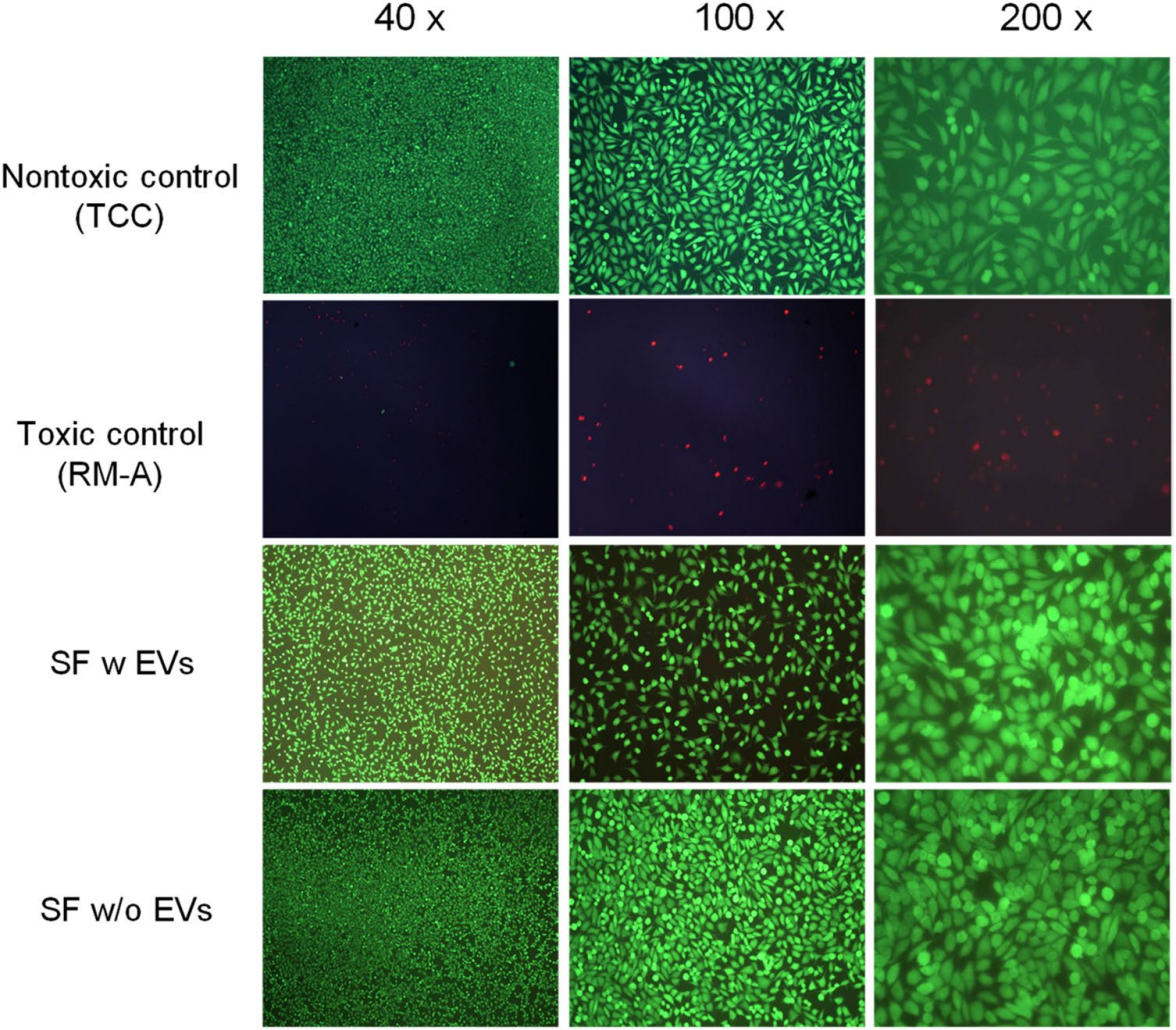
Results of the cytotoxicity testing with L929-mouse fibroblasts (live/ dead-staining) in direct contact with the fibroin membranes with (w) and without (w/o) EVs in different magnificnations (40 × ,100 × and 200 × ).

After live/dead staining with a L929-mouse fibroblast cell line, an almost equal number of vital (green stained) and well-distributed spindle-shaped cells were detected in both the SF-membranes with (w) EVs and the SF-membranes without (w/o) EVs and the negative control (Tissue Culture Coverslips, Sarstedt, Germany (TCC)). The absence of dead (red-stained) cells confirmed the assumption that no toxicity is associated with these two variants, while the toxic positive control (Polyurethan Film with 0,1% zinc diethyldithiocarbamate (ZDEC), Hatano Research Institute, Food and Drug Safety Center, Japan (RMA)) only sporadically showed cells on the tested surfaces (Fig. 8). In conclusion, both indirect and direct cytotoxicity testing indicated a good biological response of the SF-membranes, without any signs of cytotoxic potential.

## Discussion

Silk fibroin as a biomaterial of natural origin has already been used for years in the field of medical technology, among other things as a suture material^39^. The excellent biological and mechanical properties of fibroin combined with its outstanding adjustable biodegradability have made its application the focus of regenerative therapy approaches for many years^40^. Due to the advantage of the fibroin's versatile shape, a wide variety of silk shapes can be created by various techniques using electrospinning, casting, or freeze–thaw processes^40^. Especially for regenerative approaches, it is essential to generate a suitable scaffold with the aim of implanting this at the target site and to create a suitable microenvironment that closely resembles the host tissue to induce the desired cellular responses^41^. The silk fibroin structure chosen in this study represents a fibroin membrane drawn on a Poly-Tetra-Fluor-Ethylen (PTFE) plate, which combines transparent and flexible properties. Previous studies have shown that post-treatment techniques using water vapor or methanol for insolubility causes a brittle character in fibroin films^42–44^. By adding plasticizers such as sorbitol or glycerol at an early stage, such a complex post-treatment step can become obsolete and excellent flexible and transparent films can be produced, as shown by Srivastava et al.^45^. EVs are also drawing significant attention in the biomaterials field due to their distinct characteristics and broad potential applications in various biomedical areas. These membranous particles, which are enclosed by a bi-lipid membrane, carry a diverse cargo comprising proteins, lipids, nucleic acids, and other bioactive molecules. This cargo can be transferred to recipient cells, thus influencing their behavior and functions. In our study, EVs derived from an immortalized gingiva-fibroblast cell line demonstrated an anticipated size range of 120–180 nm. Multimodal protein analysis further revealed high expression of common EV markers, including CD9 and CD63, along with elevated levels of CD105, CD29, CD142, and CD44 on our EVs. These markers, associated with a range of cellular processes, underscore the diverse functions of gingiva-fibroblast EVs. The presence of CD105, CD29, CD142, and CD44 on EV surfaces suggests potential contributions to intercellular communication, tissue homeostasis, and responses to physiological cues.

A noteworthy finding is the significant abundance of CD81 on gingiva-fibroblast EVs, indicating its potential significance in facilitating interactions with target cells. This heightened presence of CD81 suggests a crucial role in intercellular communication and cargo delivery. The characteristics and molecular profile of gingiva-fibroblast EVs, as revealed by this study, highlight their potential in biomaterial applications. The multifaceted nature of their functions, coupled with the specific markers identified, suggests a promising role in influencing cellular behavior, maintaining tissue homeostasis, and responding to physiological cues. Nevertheless, it should be noted that, based on the isolation method, the group of EVs contains different subpopulations (also visible in Fig. 1A) that can carry different surface markers and can also have different biological effects in principle.

The main challenge to enable a controlled release of EVs and to keep them at the site of action for a longer period of time, if possible, can be enabled by incorporation into a suitable biomaterial. Previous studies have already shown that the integration of EVs into different carrier structures proved to be more efficient than a direct application into the respective defect^12,22,28,29,31,46^. Shi et al.^29^ for example showed that hybrid 3D scaffolds of silk and chitosan, which were loaded with EVs derived from gingival mesenchymal stem cells, exhibited improved wound healing in diabetic rats.

Interestingly, there are no published approaches on the formulation of an already EV-loaded silk fibroin solution, which is ultimately processed into a support structure e.g. in the form of a membrane. In previously published studies, only a subsequent loading of e.g. already spun fibers was performed, which does not sufficiently enable a continuous release of EVs into the wound defect during the entire degradation process of the biomaterial^47,48^. The structure of a drawn fibroin membrane used here, in which the EVs have already been incorporated into the protein solution, thus represents an innovative approach to preserving the biological native function of the EVs and, moreover to unify them with a novel biomaterial. In this study, SEM images were taken both in the top view and in cross-section in order to detect vesicles. Although the freeze-drying process was used in addition to air-dried membranes, it was not possible to detect EVs satisfactorily. This may be due on the one hand to the vanishingly small size of the vesicles, and on the other hand to the fibroin structure used in this case. Here, more macro porous structures such as 3D scaffolds may be more suitable, in which the EVs could be embed better and adhere within the pores^49,50^. Using transmission electron microscopy, it was finally possible to identify EVs within the silk membrane, according to Ricklefs et al.^38^.

Conversely, by pulling the membrane on the PTFE mould, a homogeneous distribution of the EVs in the membrane could not be achieved. A more even distribution of additives could possibly be achieved by means of further alternative processing options such as electrospinning or gas-foaming processes^49,50^. Future research should therefore investigate which fibroin structures represent a more sensible alternative as a bioactive cell carrier in order to be able to guarantee an ideal and homogeneously distributed release behaviour for subsequent application in the field of regeneration and wound healing. Additional in vitro tests were performed to further evaluate the biocompatibility of fibroin the membranes. No cytotoxic effects could be observed, neither in the functionalized membranes with EVs nor in the control group. Similar results could already be presented in previous studies from our research group^33,51^. The enhanced viability observed for L929 cells in both SF with EVs and SF without EVs compared to the toxic control group suggests a potential protective effect against the tested toxic agent. The higher viability values indicate that these samples have the ability to mitigate the toxic effects induced by the control substance. In general, these results suggest that L929 cells may be more susceptible to the toxic effects of SF w EVs and w/o EVs compared to MC3T3 cells. This interesting effect has also been described by others comparing the cytotoxic effects of different materials on these two cell lines^52–54^. Therefore, it may be beneficial to include more than one cell line in the assessment of potential cytotoxic effects of materials or medical devices. This finding suggests that SF with EVs and SF without EVs may contain compounds or factors that promote cellular survival and counteract the toxicity. These results indicate that further investigation into the composition and mechanism of action of SF with EVs and SF without EVs are required. The functionalized and non-functionalized fibroin membranes showed good proliferation in the first 5 days. From day 5, the peak is exceeded and the number of cells decrease. On the other hand, studies according to Mandal et al.^55^ show that proliferation in silk structures is particularly dependent on their porosity and pore size. In a next step, the release kinetics of the EVs from the carrier material will also be analyzed, which can presumably be adjusted by the porosity of the SF structure. Since the membrane used here is a pore-free fibroin structure, this could be a limiting factor for a more pronounced proliferation of the cells^55,56^. In addition, the test is dependent on the position of the vesicles in the membrane. However, due to the shrinking process of the membrane during drying, it is possible that some of the vesicles have attached themselves to the surface of the membrane and have a positive effect on the proliferation of the cells. Since a cell type-specific morphology and a high cell density were detected on the pure and the functionalized fibroin membranes during live/dead staining, we assume that the pure fibroin structure has a high potential for use as a bioactive material. Similar results could also be obtained from Kopp et al.^33^.

## Materials and methods

### Production of PureSilk solution

The silk fibroin aqueous solution was obtained using *PureSilk* technology (Fibrothelium GmbH, Aachen, Germany) enabling medical grade quality on an industrial scale for a broad range of concentrations. Briefly, fibroin was separated from sericin by degumming it in hot alkali solution before dissolving it in a proprietary non-toxic solvent system based on Ajisawa’s reagent. The dissolved fibroin was fully dialyzed against VE water within 8 h using tailored extraction processing. The fibroin concentration used within this study was adjusted to 3 wt% and stored at 4 °C.

### Cell culture, Isolation and Characterization of EVs

Immortalized human gingival fibroblasts (P10866, Innoprot, Derio (Bizkaia), Spain) were cultivated in α-MEM depleted of exosomes for 72 h after the last change of medium. Exosome-depleted α-MEM was produced by ultracentrifugation at 110,000 × g for 5 h. The supernatant was then accumulated as conditioned medium (CM). EVs were recovered from the CM by differential centrifugation in cell culture passages 2–7. In brief, dead cells, cell debris, and large vesicles were extracted by centrifugation at 300 × g for 10 min, 2000 × g for 20 min, and 10,000 × g for 40 min. The supernatant was pooled, and EVs were then pelleted by ultracentrifugation in an Optima LE-80 K ultracentrifuge equipped with a SW 32 Ti rotor (Beckman Coulter, Chaska, MN, USA) at 110,000 × g for 90 min. The pellet was rinsed with PBS and a second run of ultracentrifugation was performed as described above. Total protein content of EVs was measured using a BCA Protein Assay Kit (Thermo Fisher Scientific, Waltham, MA, USA). Afterwards, the EVs were analyzed by Nanoparticle Tracking Analysis (NTA) and Imaging Flow Cytometry (IFCM) analysis described in detail in Ricklefs et al.^38^.

For the phenotypical characterization of SF EVs, we used the MACSPlex Exosome kit (Miltenyi). This bead-based assay allows the simultaneous detection of 37 surface markers to determine the cellular origin of the EVs. In brief, EVs were incubated overnight with antibody-coated capture beads, washed, and incubated with tetraspanin CD9/CD81/CD63 antibodies provided in the kit. The measurements were done at FACSCanto II (BD Biosciences).

### Preparation of fibroin films

Flexible SF films were obtained by adding glycerol to 10 ml SF solution The initial quantity of EVs was 248 µg. For functionalization, the fibroin solution was priorly mixed with 24.8 µg EVs/ml SF solution (SF w| w/o EVs). The solution was then stirred for 5 min to ensure homogeneous mixture of glycerol and SF. Following that, the SF solution was casted on a PTFE mold. After drying for 24 h at 21 °C under a laminar hood, the final fibroin membrane had a thickness of approximately 200 µm bevor drying and 40 µm after drying process. The thickness was determined by a digital micrometer (Micromar 40 ER, Mahr GmbH, Germany). The films were then stored in press-seal bags at 4 °C until further use.

### Scanning electron microscopy

The surface morphology of the samples was characterized by SEM (Crossbeam 340, Zeiss, Oberkochen, Germany). Before imaging, the specimens were dried under a laminar soil hood for seven days. For cross-sectional imaging, a clear cut was made through the midline of the specimens using a scalpel. The samples were then mounted on a sample holder and subsequently coated with gold to ensure electron stability (Sputter Coater S150B, Edwards, London, UK). Imaging was accomplished in secondary electron (SE) mode at an excitation voltage of 5 kV, a scanning distance of 5 mm, and magnifications ranging from 500 ×  to 10,000 × . Subsequently, a part of the membranes was air-dried and another part was lyophilized to better detect differences under SEM.

### Transmission electron microscopy

Membranes were fixed in 3% glutaraldehyde in PBS. Samples were washed in 0.1 M Soerensen’s phosphate buffer (Merck, Darmstadt, Germany), post-fixed in 1% OsO4 (Roth, Karlsruhe, Germany) in 25 mM sucrose buffer (Merck, Darmstadt, Germany) and dehydrated by ascending ethanol series (30, 50, 70, 90 and 100%) for 10 min each. Last step was repeated 3 times. Dehydrated specimens were incubated in propylene oxide (Serva, Heidelberg, Germany) for 30 min, in a mixture of Epon resin (Serva, Heidelberg, Germany) and propylene oxide (1:1) for 1 h and finally in pure Epon for 1 h. Samples were embedded in pure Epon. Epon polymerization was performed at 90 °C for 2 h. Ultrathin sections (70–100 nm) were picked up on Cu/Rh grids (HR23 Maxtaform, Plano, Wetzlar, Germany). Contrast was enhanced by staining with 0.5% uranyl acetate and 1% lead citrate (both EMS, Munich, Germany). Samples were examined using a TEM LEO 906 (Carl Zeiss, Oberkochen, Germany), operating at an acceleration voltage of 60 kV.

### Cytocompatibility and Proliferation

In vitro cytocompatibility testing was performed as previously described^57^ using indirect viability and cytotoxicity assays. L929-mouse fibroblasts were purchased from the European Collection of Cell Culture, ECACC (Salisbury, United Kingdom). L929 mouse fibroblasts were obtained from the European Collection of Cell Culture, ECACC (Salisbury, UK). Cells were incubated in MEM supplemented with 10% foetal bovine serum, glutamine to a final concentration of 4 mM and penicillin/streptomycin (100 U/ml each) (all from Life Technologies, Carlsbad, USA), hereafter referred to as cell culture medium, at 37 °C, 5% CO2 and 95% humidity (cell culture conditions). The cells were passaged when they had achieved approximately 80% confluence. MC3T3 cells were referred from MC3T3-E1 cell line from the American Type Culture Collection (ATCC, United States of America). Cells were cultured in MEM-alpha that was supplemented with 10% fetal bovine serum and penicillin/streptomycin (100 U/ml each) (all from Life Technologies, Carlsbad, USA), hereafter referred to as cell culture medium, at 37 °C, 5% CO2 and 95% humidity (cell culture conditions). The cells were passaged when they had grown to approximately 80% confluence. Briefly, for indirect assays, three samples of each specimen type and toxic control samples were isolated under cell culture conditions (37 °C, 5% CO2, and 95% humidity) with cell culture medium at a ratio of 3 cm^2^/ml, whereas cell culture medium incubated under the same conditions functioned as a negative control. The extracts underwent centrifugation at 14.000 rpm for 10 min, and the supernatants were used as medium for cell culture for 24 h. Subsequently, the Cytotoxicity and Proliferation were measured using LDH-Assay (BioVision, Milpitas, USA) and XTT-Assay (Cell Proliferation Kit II, Roche Diagnostics, Mannheim, Germany) assay kits following the manufacturer's instructions. The cell growth on the surface of the material was observed under a microscope at two, four, six and eight days respectively. The optical density (OD) values of the supernatants were measured at 450 nm and a reference wavelength of 650 nm.

### Live-dead-staining Assay

The Assay was carried out after an incubation time of 24 h under cell culture conditions with L929-mouse fibroblasts. To stain the surfaces of the samples with live and dead cells, 60 μl per ml of medium of propidium iodide (PI) stock solution (50 μg/ml in PBS) and 500 μl per ml of medium of fresh fluorescein diacetate (FDA) working solution (20 μg/ml in PBS from 5 mg/ml FDA in acetone stock solution) were dispensed into each well.The live-dead staining assay was performed at a surface-to-volume ratio of 5.65 cm^2^/ml and the stained specimens were then visualized using an upright fluorescence microscope (Eclipse Ti-S/L100, Nikon, Düsseldorf, Germany) fitted with a red and green fluorescence parallel detection filter. Pictures were taken using a 40 × , 100 × and 200 × magnification.

### Statistics

Statistical analysis was performed using SPSS 21 (IBM, Armonk, NY, USA). The significance of differences in viability and toxicity was assessed using one-way analysis of variance (ANOVA) followed by LSD post-hoc test. **p < 0.05 indicates a significant difference for all tested groups.

## Data Availability

The datasets generated during and/or analyzed used in this manuscript are available from the corresponding author upon reasonable request.

## References

[1] Stevic I, Buescher G, Ricklefs FL (2020). Monitoring therapy efficiency in cancer through extracellular vesicles. Cells.

[2] Babuta M, Szabo G (2021). Extracellular vesicles in inflammation: Focus on the microRNA cargo of EVs in modulation of liver diseases. J. Leukoc. Biol..

[3] Batsali AK, Georgopoulou A, Mavroudi I, Matheakakis A, Pontikoglou CG, Papadaki HA (2020). The role of bone marrow mesenchymal stem cell derived extracellular vesicles (MSC-EVs) in normal and abnormal hematopoiesis and their therapeutic potential. J. Clin. Med..

[4] Del Fattore A (2015). Differential effects of extracellular vesicles secreted by mesenchymal stem cells from different sources on glioblastoma cells. Expert Opin. Biol. Ther..

[5] Zheng G (2018). Mesenchymal stromal cell-derived extracellular vesicles: Regenerative and immunomodulatory effects and potential applications in sepsis. Cell Tissue Res..

[6] Dabrowska S, Andrzejewska A, Janowski M, Lukomska B (2021). Immunomodulatory and regenerative effects of mesenchymal stem cells and extracellular vesicles: Therapeutic outlook for inflammatory and degenerative diseases. Front. Immunol..

[7] Görgens A (2022). Identification of storage conditions stabilizing extracellular vesicles preparations. J. Extracell. Vesicles.

[8] Trenkenschuh E, Richter M, Heinrich E, Koch M, Fuhrmann G, Friess W (2022). Enhancing the stabilization potential of lyophilization for extracellular vesicles. Adv. Healthc. Mater..

[9] Alcaraz MJ, Compañ A, Guillén MI (2019). Extracellular vesicles from mesenchymal stem cells as novel treatments for musculoskeletal diseases. Cells.

[10] Colao IL, Corteling R, Bracewell D, Wall I (2018). Manufacturing exosomes: A promising therapeutic platform. Trends Mol. Med..

[11] Gardiner C (2016). Techniques used for the isolation and characterization of extracellular vesicles: Results of a worldwide survey. J. Extracell. Vesicles.

[12] Li P, Kaslan M, Lee SH, Yao J, Gao Z (2017). Progress in exosome isolation techniques. Theranostics.

[13] Momen-Heravi F (2013). Current methods for the isolation of extracellular vesicles. bchm.

[14] Patel GK (2019). Comparative analysis of exosome isolation methods using culture supernatant for optimum yield, purity and downstream applications. Sci. Rep..

[15] Phinney DG, Pittenger MF (2017). Concise review: MSC-derived exosomes for cell-free therapy. Stem Cells.

[16] Ramasubramanian L, Kumar P, Wang A (2019). Engineering extracellular vesicles as nanotherapeutics for regenerative medicine. Biomolecules.

[17] Riazifar M, Pone EJ, Lötvall J, Zhao W (2017). Stem cell extracellular vesicles: Extended messages of regeneration. Annu. Rev. Pharmacol. Toxicol..

[18] Théry C (2018). Minimal information for studies of extracellular vesicles 2018 (MISEV2018): A position statement of the international society for extracellular vesicles and update of the MISEV2014 guidelines. J. Extracell. Vesicles.

[19] Willis GR, Kourembanas S, Mitsialis SA (2017). Toward exosome-based therapeutics: Isolation, heterogeneity, and fit-for-purpose potency. Front. Cardiovasc. Med..

[20] Witwer KW (2013). Standardization of sample collection, isolation and analysis methods in extracellular vesicle research. J. Extracell. Vesicles.

[21] Zhu X (2017). Comprehensive toxicity and immunogenicity studies reveal minimal effects in mice following sustained dosing of extracellular vesicles derived from HEK293T cells. J. Extracell. Vesicles.

[22] Zhang S (2020). Extracellular vesicles-loaded fibrin gel supports rapid neovascularization for dental pulp regeneration. Int. J. Mol. Sci..

[23] Liu Y (2021). Exosomes derived from stem cells from apical papilla promote craniofacial soft tissue regeneration by enhancing Cdc42-mediated vascularization. Stem Cell Res. Ther..

[24] Qian Z (2020). A moisturizing chitosan-silk fibroin dressing with silver nanoparticles-adsorbed exosomes for repairing infected wounds. J. Mater. Chem. B.

[25] Xian X, Gong Q, Li C, Guo B, Jiang H (2018). Exosomes with highly angiogenic potential for possible use in pulp regeneration. J. Endod..

[26] Huang C-C, Narayanan R, Alapati S, Ravindran S (2016). Exosomes as biomimetic tools for stem cell differentiation: Applications in dental pulp tissue regeneration. Biomaterials.

[27] Wiklander OPB (2015). Extracellular vesicle in vivo biodistribution is determined by cell source, route of administration and targeting. J. Extracell. Vesicles.

[28] Han C (2019). Delivery of miR-675 by stem cell-derived exosomes encapsulated in silk fibroin hydrogel prevents aging-induced vascular dysfunction in mouse hindlimb. Mater. Sci. Eng. C.

[29] Shi Q (2017). GMSC-derived exosomes combined with a chitosan/silk hydrogel sponge accelerates wound healing in a diabetic rat skin defect model. Front. Physiol..

[30] Liu X (2017). Integration of stem cell-derived exosomes with in situ hydrogel glue as a promising tissue patch for articular cartilage regeneration. Nanoscale.

[31] Xu N (2018). Wound healing effects of a Curcuma zedoaria polysaccharide with platelet-rich plasma exosomes assembled on chitosan/silk hydrogel sponge in a diabetic rat model. Int. J. Biol. Macromol..

[32] Patil PP, Reagan MR, Bohara RA (2020). Silk fibroin and silk-based biomaterial derivatives for ideal wound dressings. Int. J. Biol. Macromol..

[33] Kopp A (2020). Effect of process parameters on additive-free electrospinning of regenerated silk fibroin nonwovens. Bioact. Mater..

[34] Kopp A (2019). Production and characterization of porous fibroin scaffolds for regenerative medical application. In Vivo (Brooklyn).

[35] Chouhan D, Mandal BB (2020). Silk biomaterials in wound healing and skin regeneration therapeutics: From bench to bedside. Acta Biomater..

[36] Thangavel P, Ramachandran B, Kannan R, Muthuvijayan V (2017). Biomimetic hydrogel loaded with silk and <scp>l</scp> -proline for tissue engineering and wound healing applications. J. Biomed. Mater. Res. Part B Appl. Biomater..

[37] Buser D, Brägger U, Lang NP, Nyman S (1990). Regeneration and enlargement of jaw bone using guided tissue regeneration. Clin. Oral Implants Res..

[38] Ricklefs FL (2019). Imaging flow cytometry facilitates multiparametric characterization of extracellular vesicles in malignant brain tumours. J. Extracell. Vesicles.

[39] Tajirian AL, Goldberg DJ (2010). A review of sutures and other skin closure materials. J. Cosmet. Laser Ther..

[40] Chen K, Li Y, Li Y, Pan W, Tan G (2023). Silk fibroin combined with electrospinning as a promising strategy for tissue regeneration. Macromol. Biosci..

[41] Kundu B, Rajkhowa R, Kundu SC, Wang X (2013). Silk fibroin biomaterials for tissue regenerations. Adv. Drug Deliv. Rev..

[42] Chen C, Chuanbao C, Xilan M, Yin T, Hesun Z (2006). Preparation of non-woven mats from all-aqueous silk fibroin solution with electrospinning method. Polymer (Guildf).

[43] Ha SW, Tonelli AE, Hudson SM (2005). Structural studies of Bombyx mori silk fibroin during regeneration from solutions and wet fiber spinning. Biomacromolecules.

[44] Bayraktar O, Malay O, Ozgarip Y, Batigun A (2005). Silk fibroin as a novel coating material for controlled release of theophylline. Eur. J. Pharm. Biopharm..

[45] Srivastava CM, Purwar R, Kannaujia R, Sharma D (2015). Flexible silk fibroin films for wound dressing. Fibers Polym..

[46] Song Y (2022). Silk sericin patches delivering miRNA-29-enriched extracellular vesicles-decorated myoblasts (SPEED) enhances regeneration and functional repair after severe skeletal muscle injury. Biomaterials.

[47] Cunnane EM (2022). Extracellular vesicles enhance the remodeling of cell-free silk vascular scaffolds in rat aortae. AC S Appl. Mater. Interfaces.

[48] Kyung Kim D, Lee S, Kim M, Jeong Y, Lee S (2021). Exosome-coated silk fibroin 3D-scaffold for inducing osteogenic differentiation of bone marrow derived mesenchymal stem cells. Chem. Eng. J..

[49] Wang Z (2022). Engineered multifunctional Silk fibroin cryogel loaded with exosomes to promote the regeneration of annulus fibrosus. Appl. Mater. Today.

[50] Zhang X, Cao C, Ma X, Li Y (2012). Optimization of macroporous 3-D silk fibroin scaffolds by salt-leaching procedure in organic solvent-free conditions. J. Mater. Sci. Mater. Med..

[51] Kopp A (2020). Influence of the casting concentration on the mechanical and optical properties of Fa/CaCl2-derived silk fibroin membranes. Int. J. Mol. Sci..

[52] Shin H, Ko H, Kim M (2016). Cytotoxicity and biocompatibility of Zirconia (Y-TZP) posts with various dental cements. Restor. Dent. Endod..

[53] Wang MO, Etheridge JM, Thompson JA, Vorwald CE, Dean D, Fisher JP (2013). Evaluation of the in vitro cytotoxicity of cross-linked biomaterials. Biomacromolecules.

[54] Brackett MG (2010). Cytotoxic response of three cell lines exposed in vitro to dental endodontic sealers. J. Biomed. Mater. Res. Part B Appl. Biomater..

[55] Mandal BB, Kundu SC (2009). Cell proliferation and migration in silk fibroin 3D scaffolds. Biomaterials.

[56] Jiang S (2021). Effects of different aperture-sized type I collagen/silk fibroin scaffolds on the proliferation and differentiation of human dental pulp cells. Regen. Biomater..

[57] Jung O (2019). Improved in vitro test procedure for full assessment of the cytocompatibility of degradable magnesium based on ISO 10993-5/-12. Int. J. Mol. Sci..

